# Difference in Macroscopic Morphologies of Amylosic Supramolecular Networks Depending on Guest Polymers in Vine-Twining Polymerization

**DOI:** 10.3390/polym10111277

**Published:** 2018-11-16

**Authors:** Saya Orio, Takuya Shoji, Kazuya Yamamoto, Jun-ichi Kadokawa

**Affiliations:** Department of Chemistry, Biotechnology, and Chemical Engineering, Graduate School of Science and Engineering, Kagoshima University, 1-21-40 Korimoto, Kagoshima 860-0065, Japan; k3951278@kadai.jp (S.O.); k6911901@kadai.jp (T.S.); yamamoto@eng.kagoshima-u.ac.jp (K.Y.)

**Keywords:** amylose, gelation, guest polymer, supramolecular network, vine-twining polymerization

## Abstract

Amylose, a natural polysaccharide, acts as a host molecule to form supramolecular inclusion complexes in its enzymatically formation process, that is, phosphorylase-catalyzed enzymatic polymerization using the α-d-glucose 1-phosphate monomer and the maltooligosaccharide primer, in the presence of appropriate guest polymers (vine-twining polymerization). Furthermore, in the vine-twining polymerization using maltooligosaccharide primer-grafted polymers, such as maltoheptaose (G_7_)-grafted poly(γ-glutamic acid) (PGA), in the presence of poly(ε-caprolactone) (PCL), the enzymatically elongated amylose graft chains have formed inclusion complexes with PCL among the PGA main-chains to construct supramolecular networks. Either hydrogelation or aggregation as a macroscopic morphology from the products was observed in accordance with PCL/primer feed ratios. In this study, we evaluated macroscopic morphologies from such amylosic supramolecular networks with different guest polymers in the vine-twining polymerization using G_7_-grafted PGA in the presence of polytetrahydrofuran (PTHF), PCL, and poly(l-lactide) (PLLA). Consequently, we found that the reaction mixture using PTHF totally turned into a hydrogel form, whereas the products using PCL and PLLA were aggregated in the reaction mixtures. The produced networks were characterized by powder X-ray diffraction and scanning electron microscopic measurements. The difference in the macroscopic morphologies was reasonably explained by stabilities of the complexes depending on the guest polymers.

## 1. Introduction

Polysaccharides and polypeptides, the major classes of biopolymers, exhibit specific biological functions in nature, in accordance with their controlled primary structures, as well as regular higher-order structures [1,2,3]. For example, amylose, which is a natural polysaccharide composed of glucose (G) repeating units, is known to form a regularly controlled double helical assembly in water [4,5], owing to α(1 → 4)-glycosidic arrangement in the G chain. Furthermore, hybrid systems from several kinds of biopolymers are also present in nature, such polysaccharide-protein (or peptide) conjugates as proteoglycans/peptidoglycans or glycoproteins [6,7,8,9]. Accordingly, artificial saccharide-peptide conjugates can be expected as new bio-related functional materials, which have a potential for practical applications in biomedical and tissue engineering fields.

Because biopolymers are composed of a wide variety of unit structures linked through controlled arrangements and contain functional groups such as hydroxy, amino, and carboxy groups, their chemical synthesis with well-defined structure generally requires multiple procedures. Particularly, as polysaccharides have very complicated structures compared with other biopolymers, such as polypeptides, owing to comprising a variety of monosaccharide residues linked through regio- and stereocontrolled glycosidic linkages, it is difficult to exploit general organic reactions for their chemical synthesis with highly controlled fashions. As an alternative to such an organic reaction method, the enzymatic approach has been identified as a very powerful tool to efficiently synthesize well-defined polysaccharides by simple operations [10,11,12,13,14]. For example, amylose with well-defined structure can be synthesized by phosphorylase-catalyzed enzymatic polymerization using α-d-glucose 1-phosphate (G-1-P) and maltooligosaccharide as a monomer and a primer, respectively [15,16,17,18,19]. The polymerization is initiated from the nonreducing end of the maltooligosaccharide primer and G residues are consecutively transferred from G-1-P to the propagating nonreducing end with liberating inorganic phosphate (Pi) by the enzymatic catalysis, according to the following reaction manner: [α-(1 → 4)-G]*_n_* + G-1-P ⇄ [α-(1 → 4)-G]*_n+_*_1_ + Pi. Owing to no participation of the other end side of the maltooligosaccharide primer, that is, the reducing end, into the reaction, the phosphorylase-catalyzed enzymatic polymerization also progresses from a primer-grafted polymer, where the reducing ends of the plural maltooligosaccharide primers are covalently attached on the polymeric chain, to produce an amylose-grafted polymer [13,14,18,19,20,21,22,23,24]. In the previous study using such a primer-grafted substrate, for example, we investigated synthesis of an amylose-grafted poly(γ-glutamic acid) (PGA) as an artificial saccharide-peptide conjugate [25], because PGA is a well-known natural polypeptide [26,27]. In the reaction media, the elongated amylose graft chains spontaneously formed double helical cross-linking points among the PGA main-chains, resulting in the formation of a hydrogel.

Besides the double helical formation, amylose is known as a host molecule to form supramolecular inclusion complexes with hydrophobic guest molecules having suitable molecular shapes and chain lengths, such as fatty acids and fatty alcohols, by hydrophobic interaction [28]. We have reported the formation of amylosic supramolecular inclusion complexes with polymeric guest molecules by means of the propagating process in the phosphorylase-catalyzed enzymatic polymerization of the G-1-P monomer with the maltoheptaose (G_7_) primer [20,23,29,30,31,32,33]. When the enzymatic polymerization is conducted in the presence of suitable hydrophobic polymers, such as poly(ε-caprolactone) (PCL), polytetrahydrofuran (PTHF), and poly(l-lactide) (PLLA), dispersed in aqueous buffer solution as polymerization solvent, the propagating amylose chain helically interacts with the polymers with the progress of the reaction to form inclusion complexes. As the representation of the propagation in this system resembles the way that vines of a plant grow, twining around a rod, we have proposed that this polymerization method to construct amylose-polymer inclusion complexes is named “vine-twining polymerization” [20,23,29,30,31,32,33]. In the following investigations, we evaluated the stability of amylose–polymer inclusion complexes with the different guest polymers under solution state in dimethyl sulfoxide (DMSO). The analytical results of the investigation indicated that stability in the DMSO solutions increased in accordance with the bulkiness of the guest polymers as follows: PTHF < PCL < PLLA [34].

**Figure 1:**
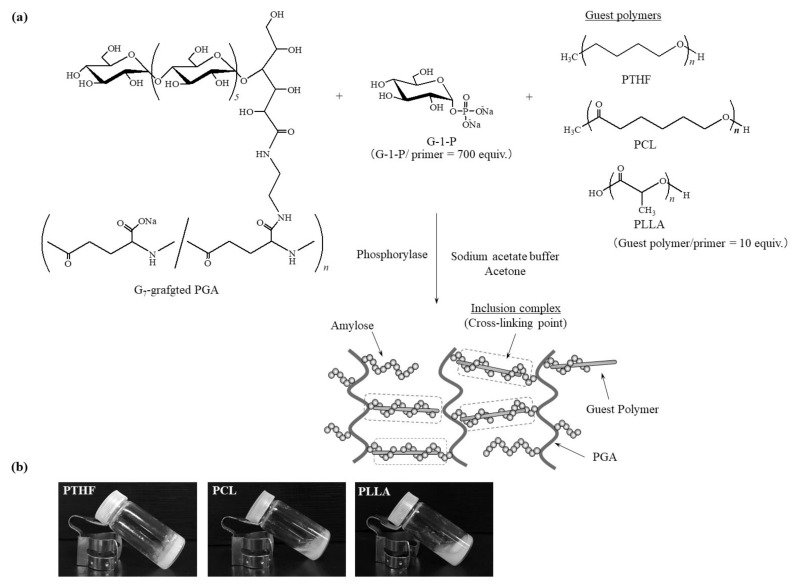
Image for construction of supramolecular networks by vine-twining polymerization using G_7_-grafted poly(γ-glutamic acid) (PGA) in the presence of polytetrahydrofuran (PTHF), poly(ε-caprolactone) (PCL), and poly(l-lactide) (PLLA) (**a**) and photographs of reaction mixtures from three guest polymers (**b**).

Furthermore, we also attempted to fabricate supramolecular networks composed of amylose-PCL inclusion complexes as cross-linking points by the vine-twining polymerization in the presence of PCL using G_7_-grafted PGA instead of the pure G_7_ as the primer [35]. It was a consequence that PCL/primer feed ratios were a key factor whether the resulting networks formed hydrogels or aggregates as macroscopic morphologies from the network products. The predominant formation of double helixes from the enzymatically elongated amylose graft chains in the products in the presence of lower ratios of PCL led to the hierarchical construction of larger macroscopic networks, resulting in hydrogelation of the reaction mixtures. In the presence of higher ratios of PCL, on the other hand, smaller networks mostly composed of amylose-PCL inclusion complexes were formed according to the vine-twining polymerization manner, giving rise to the production of aggregates in the reaction mixtures. In this study, we evaluated macroscopic morphologies from the amylosic supramolecular networks with the different guest polymers by the vine-twining polymerization using G_7_-grafted PGA in the presence of the guest polymers, PTHF, PCL, and PLLA (Figure 1a). Consequently, we found that the reaction mixture using PTHF totally turned into a hydrogel form, whereas the products using PCL and PLLA were aggregated in the reaction mixtures (Figure 1b).

## 2. Materials and Methods

### 2.1. Materials

PGA (M.W. = 1.5–2.5 × 10^6^) was purchased from Wako Pure Chemicals, Tokyo, Japan. Thermostable phosphorylase from *Aquifex aeolicus* VF5 was supplied by Ezaki Glico Co., Ltd., Osaka, Japan [17,36,37]. G_7_-grafted PGA was synthesized according to the literature procedure [25]; ^1^H NMR (D_2_O) δ 1.86-2.01, 2.01-2.18 (br, β-CH_2_ of PGA), 2.30-2.42 (br, γ-CH_2_ of PGA), 3.40–4.08 (br, sugar protons of H2-H6), 4.12-4.25 (br, α-CH of PGA), 5.18, 5.41 (br s, H1 of G_7_). The degree of substitution (DS) for the grafting was determined by the integrated ratio of the H1 signal of G_7_ to the γ-CH_2_ signal of PGA to be 70.2%. The guest polymers, PTHF, PCL, and PLLA, were synthesized by ring-opening polymerization of THF, CL, and LLA monomers initiated with methyl trifluoromethanesulfonate, 6-hydroxycaproic acid, and l-lactic acid, respectively [38,39,40]. The *M*_n_ values were calculated by the integrated ratios of the main-chain signals to the terminal signals to be 2190, 1200, and 1370, respectively. Other reagents and solvents were available commercially and used without further purification.

### 2.2. Vine-Twining Polymerization Using G_7_-grafted PGA in the Presence of PTHF

G_7_-grafted PGA (0.0013 g, 0.0014 unit mmol, G_7_; 1.0 µmmol) was dissolved in an aqueous sodium acetate buffer solution (0.2 mol/L, pH 6.2, 1.2 mL) and G-1-P disodium salt (0.21 g, 0.7 mmol, 700 equiv. with primer) was added to the solution. After a solution of PTHF (0.022 g, 10.0 µmmol, 10 equiv. with primer) in acetone (0.06 mL) was mixed, thermostable phosphorylase (25.2 units) was added to this mixture, which was then maintained at 45 °C for 48 h. The resulting hydrogel was soaked and washed with water and acetone in turn several times for purification (0.572 g). The purified hydrogel was lyophilized to give the product (0.0722 g). 

### 2.3. Vine-Twining Polymerization Using G_7_-grafted PGA in the Presence of PCL

G_7_-grafted PGA (0.0013 g, 0.0014 unit mmol, G_7_; 1.0 µmmol) was dissolved in an aqueous sodium acetate buffer solution (0.2 mol/L, pH 6.2, 1.2 mL) and G-1-P disodium salt (0.21 g, 0.7 mmol, 700 equiv. with primer) was added to the solution. After a solution of PCL (0.012 g, 10.0 µmmol, 10 equiv. with primer) in acetone (0.06 mL) was mixed, thermostable phosphorylase (25.2 units) was added to this mixture, which was then maintained at 45 °C for 48 h. The reaction solution was then subjected to centrifugation, and washed with water and acetone in turn, several times for purification, which was then lyophilized to give the aggregate product (0.0702 g).

### 2.4. Vine-Twining Polymerization Using G_7_-grafted PGA in the Presence of PLLA

G_7_-grafted PGA (0.0013 g, 0.0014 unit mmol, G_7_; 1.0 µmmol) was dissolved in an aqueous sodium acetate buffer solution (0.2 mol/L, pH 6.2, 1.2 mL) and G-1-P disodium salt (0.21 g, 0.7 mmol, 700 equiv. with primer) was added to the solution. After a solution of PLLA (0.014 g, 10.0 µmmol, 10 equiv. with primer) in acetone (0.06 mL) was mixed, thermostable phosphorylase (25.2 units) was added to this mixture, which was then maintained at 45 °C for 48 h. The reaction solution was then subjected to centrifugation, and washed with water and acetone in turn, several times for purification, which was then lyophilized to give the aggregate product (0.0726 g).

### 2.5. Measuremsnts

The ^1^H NMR spectra were recorded using a JEOL ECX400 spectrometer (JEOL, Akishima, Tokyo, Japan). The powder X-ray diffraction (XRD) measurements were performed using a PANalytical X’Pert Pro MPD diffractometer (PANalytical B.V., EA Almelo, The Netherlands) with Ni-filtered Cu Kα radiation (λ = 0.15418 nm). The scanning electron microscopic (SEM) images were obtained using a Hitachi S-4100H electron microscope (Hitachi High-Technologies Corporation, Tokyo, Japan).

## 3. Results and Discussion

As we already reported, the macroscopic morphologies from the supramolecular networks produced by the vine-twining polymerization using G_7_-grafted PGA in the presence of PCL were dependent on the PCL/primer (G_7_) feed ratios [35]. In the presence of high feed ratios of PCL (e.g., 10 equiv. with primer), the aggregates, which were largely composed of amylose-PCL inclusion complexes, were formed in the reaction mixtures. Therefore, to evaluate the macroscopic morphologies from the amylosic supramolecular networks with the different guest polymers, that is, PTHF, PCL, and PLLA, by the vine-twining polymerization using G_7_-grafted PGA, in this study, the condition of the guest polymer/primer feed ratio = 10 was employed for the predominant formation of the inclusion complexes (Figure 1a). The vine-twining polymerization was carried out by the phosphorylase-catalyzed enzymatic polymerization in the acetate buffer dispersion of the guest polymer under the conditions of G-1-P/primer feed ratio = 700 at 45 °C for 48 h. When PLLA was used, an aggregate was produced in the reaction mixture, which was the same result by using PCL as previously reported by us (Figure 1b) [35]. On the other hand, the same operation using PTHF gave rise to hydrogelation of the reaction mixture (Figure 1b). Water content of the hydrogel was calculated based on the weight difference before and after lyophilization to be 87.4%. The monomer conversions, *M*_n_ values of amylose graft chains, and amylose contents in the products from the three guest polymers, which were estimated based on the weights after lyophilization, were comparable in all cases (ca. 50%, 60,000, and 85%).

**Figure 2:**
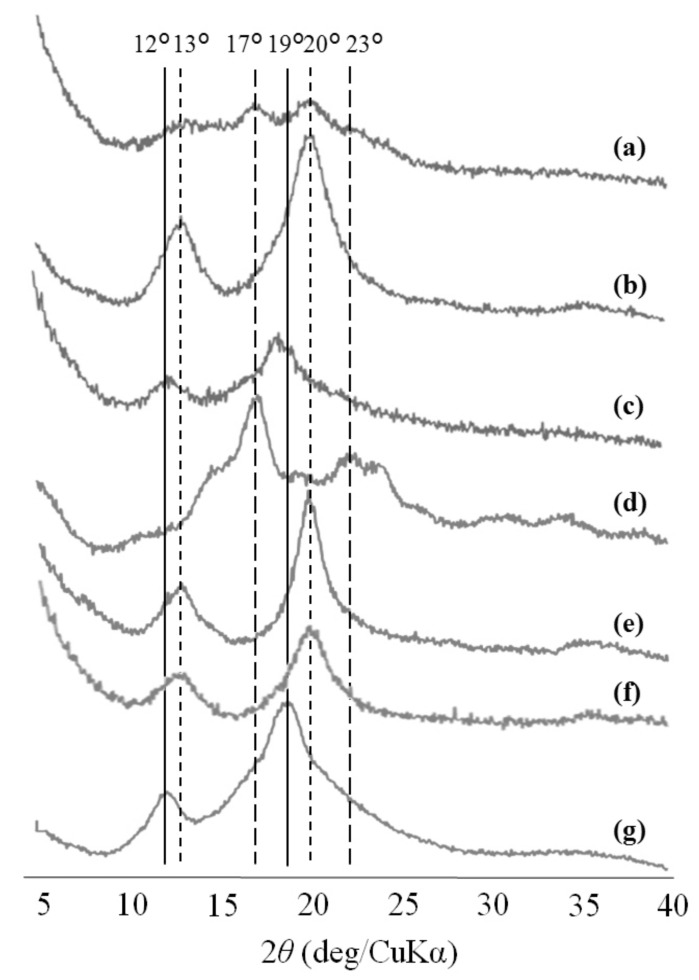
X-ray diffraction (XRD) profiles of lyophilized samples from PTHF (**a**), PCL (**b**), and PLLA (**c**), enzymatically synthesized amylose (**d**), amylose-PTHF inclusion complex (**e**), amylose-PCL inclusion complex (**f**), and amylose-PLLA inclusion complex (**g**).

To investigate further the difference in the produced supramolecular networks depending on guest polymers, the XRD measurement of lyophilized samples from the products was conducted (Figure 2). The XRD profiles of the samples from PCL and PLLA show the typical diffraction peaks at 13° and 20°, and at 12° and 19°, assignable to inclusion complexes with 6_1_ and 7_1_ helixes, respectively (Figure 2b,c), which are identical to those of the general amylose-PCL and –PLLA inclusion complexes (Figure 2f,g). On the other hand, the XRD profile of the sample from PTHF exhibits the diffraction peaks at 13° and 20°, and at 17° and 23°, ascribable to both inclusion complex and double helix, respectively (Figure 1a), which are also identical with those of the general amylose-PTHF inclusion complex and the pure amylose (Figure 1e,d). The presence of double helixes besides inclusion complexes as cross-linking points probably contributed to construct a larger network than that solely composed of inclusion complexes, leading to the macroscopic formation of the hydrogel. The similar hydrogelation from the supramolecular network comprising both double helixes and inclusion complexes was observed when a lower PCL/primer feed ratio (=3) was employed in the vine-twining polymerization using G_7_-grafted PGA [35].

**Figure 3:**
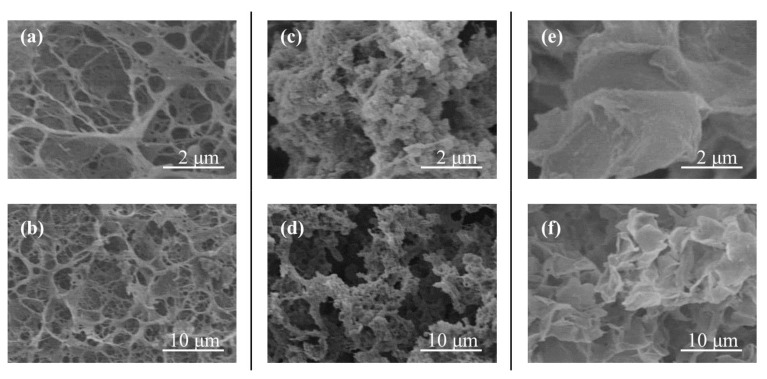
Scanning electron microscopic (SEM) images of lyophilized samples from PTHF (**a**,**b**), PCL (**c**,**d**), and PLLA (**e**,**f**).

The lyophilized samples were then subjected to SEM measurement to estimate the difference in macroscopic network sizes (Figure 3), because macroscopic morphology of the sample by lyophilization had been constructed by representatively reflecting its molecular structure, as we already reported [25,35]. Compared with the network morphology of the sample from PTHF (Figure 3a,b), the SEM images of the other two samples show the smaller network morphologies. Furthermore, network size of the sample from PLLA (Figure 3e,g) is smaller than that of the sample from PCL (Figure 3c,d). The larger network from PTHF, comprising both double helixes and inclusion complexes, has an ability to include the sufficient amounts of water for the formation of the hydrogel, whereas the smaller network, largely composed of inclusion complexes, does not exhibit an ability to retain the large amounts of water sufficiently, resulting in no hydrogelation.

**Figure 4:**
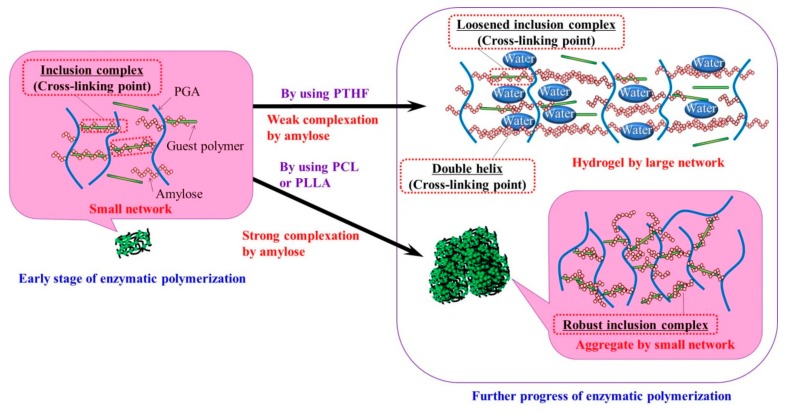
Image for formation of supramolecular networks with different sizes in accordance with guest polymers in vine-twining polymerization.

The difference in the macroscopic morphologies from the supramolecular network products by the vine-twining polymerization using PTHF, PCL, and PLLA can be explained in accordance with stabilities of the corresponding inclusion complexes as follows (Figure 4). When the phosphorylase-catalyzed enzymatic polymerization using G_7_-grafted PGA is carried out in the presence of sufficient amounts of the guest polymers (10 equiv. with primer), inclusion complexes of the elongated amylose chains with the guest polymers among the PGA main-chains are predominantly formed at the early stage of the polymerization. The inclusion complexes with the relatively low molecular weight guest polymers (*M*_n_ values = 1200–2200) act as cross-linking points to construct small network structures. Because the complexation with PCL and PLLA are stronger than that with PTHF under solution state, as we have previously reported [34], the robust inclusion complexes with PCL and PLLA remain intact during the further enzymatic polymerization, producing macroscopic aggregates from the small supramolecular networks in the reaction mixtures. Owing to the difference in stabilities of the complexes from PCL and PLLA, macroscopic network sizes of the aggregates are slightly different as observed in the SEM images (Figure 3). Because of the weak complexation behavior with PTHF under solution state, on the other hand, dissociation of complexes partly occurs during the further enzymatic polymerization. Accordingly, the dissociated amylose graft chains spontaneously form double helixes among the PGA main-chains as cross-linking points without the relatively low molecular weight guest polymers, which form the larger supramolecular network, for the macroscopic hydrogelation. During the formation of double helixes, the remaining inclusion complexes are probably loosened to also act as cross-linking points in the large supramolecular network.

## 4. Conclusions

In this study, we found that the supramolecular networks produced by the vine-twining polymerization using G_7_-grafted PGA macroscopically constructed the hydrogel or aggregate depending on the guest polymers for complexation with amylose graft chains as cross-linking points. The difference was owing to the stabilities of inclusion complexes with the respective guest polymers under solution state. The inclusion complexes with PCL and PLLA were robust during the enzymatic polymerization to construct the smaller supramolecular networks for the macroscopic aggregation of the products. On the other hand, the relatively unstable inclusion complexes with PTHF were partly dissociated under solution state during the enzymatic polymerization. The dissociated amylose graft chains spontaneously formed double helixes among the PGA main-chains to macroscopically construct the larger supramolecular network, which retained the sufficient amounts of water for hydrogelation. This study revealed that because the enzymatically produced amylose formed the different types of assemblies, that is, double helix and inclusion complex, and exhibited the different complexation stabilities depending on the guest polymers, the unique properties for the formation of macroscopic morphologies from the supramolecular networks were realized.
